# What to Look for in Red Light Therapy: A Product Guide Backed by Science

**DOI:** 10.7759/cureus.105378

**Published:** 2026-03-17

**Authors:** Colby V Spongberg, Emily R Stack, Caroline Aprigliano, Daniela Grinis, Stephanie Sawicki, Michelle Elway

**Affiliations:** 1 College of Medicine, Touro College of Osteopathic Medicine, Great Falls, USA; 2 College of Medicine, William Carey University College of Osteopathic Medicine, Hattiesburg, USA; 3 College of Osteopathic Medicine, Touro College of Osteopathic Medicine, Great Falls, USA

**Keywords:** atp production, collagen synthesis, cytochrome c oxidase, mitochondrial function, near-infrared light, nitric oxide, photobiomodulation, reactive oxygen species, red light therapy, skin rejuvenation

## Abstract

Photobiomodulation (PBM) is an emerging non-invasive modality for skin rejuvenation that utilizes specific wavelengths of red and near-infrared (NIR) light to enhance mitochondrial function and promote cellular repair. The downstream effects of mitochondrial efficiency improve wound healing, pain relief, and aesthetic outcomes. Although early research has encouraging results, the exact pathways by which PBM exerts its effects are still under investigation. PBM is known by other names, including red light therapy and low-level light therapy. Consumer devices are available in many forms, such as masks, wands, hand-held panels, laser caps, and full-body systems. With the rise in popularity, understanding the underlying biological mechanisms of PBM is becoming increasingly important. Equally critical is the selection of a product that has undergone the necessary U.S. Food and Drug Administration (FDA) channels, ensuring the device is backed by scientific evidence. This paper specifically serves as a guide for selecting safe, evidence-based PBM mask devices. This review discusses the mitochondrial and enzymatic mechanisms of action while providing a comprehensive literature review of available mask products that are both FDA-cleared and supported by scientific papers.

## Introduction and background

Photobiomodulation (PBM) is an emerging non-invasive modality for skin rejuvenation that uses specific wavelengths of red and near-infrared (NIR) light to enhance mitochondrial function and promote cellular repair. The effects of improved mitochondrial efficiency support enhanced wound healing, pain reduction, and aesthetic outcomes [1,2]. Although early research has yielded encouraging results, the exact pathways by which PBM exerts its effects remain under investigation. PBM is known by other names, including red light therapy (RLT) or low-level light therapy. Consumer devices are available in many forms, such as masks, wands, handheld panels, laser caps, and full-body systems. With the rise in popularity, understanding the underlying mechanisms of PBM is becoming increasingly important. Equally critical is the selection of a product that has undergone the necessary U.S. Food and Drug Administration (FDA) channels, ensuring the device is backed by scientific evidence. 

This paper specifically serves as a guide for selecting safe, evidence-based PBM mask devices. This review discusses the mitochondrial and enzymatic mechanisms of action while providing a comprehensive literature review of available mask products that are both FDA-cleared and supported by scientific papers. While PBM has been increasingly studied for dermatologic and other aesthetic applications, the rapid growth of consumer-usable devices, specifically LED masks, has outpaced standardized clinical guidance. This review, therefore, aims to synthesize clinical outcomes, safety considerations, and regulatory pathways to assist physicians and consumers in evaluating evidence-based PBM mask devices. 

A comprehensive narrative literature review was conducted to evaluate the mechanistic, clinical, and regulatory evidence supporting RLT mask devices. Relevant studies were identified through searches of PubMed/MEDLINE, the Cochrane Library, Embase, Google Scholar, and ClinicalTrials.gov. Search results were screened for randomized controlled trials, prospective clinical studies, and mechanistic investigations, as well as any device-specific outcome data. Regulatory information was gathered directly through the FDA 510(k) database and AccessGUDID.gov to verify device classification, product codes, and clearance status. Devices identified under relevant FDA product codes were then cross-referenced with the published literature by device name and manufacturer to assess the extent of available peer-reviewed evidence.

## Review

Mechanism of action

The most commonly cited mechanisms of photobiomodulation involve photon absorption by mitochondrial chromophores, leading to alterations in cellular metabolism. These photons are absorbed by cytochrome c oxidase (CcO), which upregulates the enzyme’s activity and increases the efficiency of the electron transport chain (ETC) [1,2]. CcO upregulation is hypothesized to occur through photodissociation of nitric oxide (NO) from the enzyme complex [3]. NO competes with oxygen on CcO, causing an inhibitory effect on the ETC [4-7]. By removing this inhibitory effect, the mitochondria are more efficient, leading to a number of downstream effects, including adenosine triphosphate (ATP) production, reactive oxygen species (ROS) production, and signal transduction. Alternative mechanisms have been suggested, including biophoton production, mechanotransduction, and photophysical mechanisms [8,9]. 

The effects of light-emitting diode (LED) therapy on CcO activity were explored by Wong-Riley et al. in multiple studies, including in vitro and in vivo trials. In the in vitro trials, cortical neurons were inhibited by potassium cyanide (KCN), an irreversible inhibitor of CcO. LED exposure restored ATP levels toward baseline under low KCN concentrations (10 μm) but not at higher concentrations (10-100 μm). In neurons exposed to tetrodotoxin, an indirect inhibitor of the enzyme, the LED completely restored control levels of enzyme activity. Continued LED treatment of tetrodotoxin-inhibited neurons upregulated CcO enzymatic activity above control levels. In vivo, rats intoxicated with methanol developed retinal dysfunction attributed to the toxic effects of formic acid on CcO. LED therapy showed significant improvement in retinal function measured by electroretinographic response [10,11].

Maghfour et al. explain the proposed downstream effects of PBM in their overview of photobiomodulation published in 2024 while addressing the need for further research to clarify the exact pathways [12]. The effects include increased ATP production, leading to cellular proliferation and differentiation, NO release, ROS production, and intracellular calcium signaling. Small increases in NO promote angiogenesis and protection from ROS, while large increases can cause damage [13-22]. While high levels of ROS are damaging, low levels appear to be beneficial by altering secondary messengers, resulting in fibroblast, collagen, and cellular proliferation and migration [23-25].

Photobiomodulation therapy most commonly uses red light (∼630-660 nm) and NIR light (∼830-850 nm). The primary difference lies in the depth of tissue penetration and in the specific cells and structures affected. Red light is absorbed more superficially, primarily affecting epidermal and upper dermal layers, which leads to benefits such as collagen production, improved skin tone, and reduction in fine lines. In contrast, NIR light penetrates deeper into the dermis and subcutaneous tissues, influencing vascular endothelial cells, muscle, and connective tissue, which enhances microcirculation, reduces inflammation, and supports deeper tissue healing [26]. 

On a physiologic level, red and NIR light differ in both cellular targets and resulting biological effects. Red light is primarily absorbed by mitochondrial chromophores such as CcO, leading to enhanced ATP production, activation of transcription factors, and stimulation of collagen synthesis [27]. In contrast, NIR light interacts more with the cell membrane, leading to downstream effects such as enhanced fibroblast-to-myofibroblast transformation, mast cell degranulation, increased phagocytic activity, and chemotaxis of immune cells [28]. These distinctions highlight how red light supports surface-level skin rejuvenation, while NIR light promotes deeper tissue repair and immunomodulation. Although both wavelengths activate similar intracellular pathways, the effects are modulated by penetration depth and the cell populations targeted. Two randomized controlled trials concluded that the combination of red and NIR wavelengths provides synergistic benefits, enhancing collagen density, reducing inflammation, and improving skin texture as well as appearance more effectively than either wavelength alone [29]. 

There is a biphasic dose response noted with PBM, which means that light therapy works better when delivered at low doses as compared to the exact same wavelength at a higher dose. Factors such as excessive ROS, NO, and the initiation of cytotoxic pathways actually act to reduce the beneficial effects of PBM [30-32]. This phenomenon has been consistently demonstrated across multiple PBM studies and stresses the importance of studying parameters such as irradiance (mW/cm²), fluence (J/cm²), pulse structure, and treatment duration. Typical irradiance levels for dermatologic applications range from 20 to 60 mW/cm² with fluences between 4 and 18 J/cm² [33-35]. Irradiance levels indicate how strong the emitting light is and the fluence describes how much light energy the skin is actually receiving. It is important to note that this wide range of irradiance levels in dosing across published trials indicates the major lack of standardization among the dosing protocols. Devices that do not disclose irradiance or fluence may operate outside the therapeutic range, therefore indicating they may not even have a clinical benefit. Future research needs to be directed toward establishing those correct dose amounts to guide both consumer and clinician device selection. 

For consumers, these mechanistic insights are not just academic. These values and pathways directly inform individuals of how their devices should be evaluated. It is important to look for masks that specify the correct wavelengths needed for absorption, report irradiance and fluence within the therapeutic window, and limit session time to avoid exceeding the biphasic dose response. Devices that lack any of these specifications or even rely on purely cosmetic marketing claims may not meaningfully engage the signaling pathways described in this section and possibly have not been evaluated correctly.

Clinical use and safety

Clinical Applications

Beyond mechanistic pathways, photobiomodulation has been evaluated across a wide range of clinical contexts, with variable study designs, treatment parameters, and reported outcomes. The benefits of PBM have begun to make their way into several clinical utilities across aesthetic outcomes. There are multiple studies indicating the benefits of PBM for skin rejuvenation and anti-aging. PBM stimulates fibroblast proliferation and induces dermal extracellular matrix remodeling. This mechanism is associated with increased collagen density, enhanced elasticity, reduced wrinkle depth, and improved overall skin texture [26,34-41]. PBM also acts to accelerate wound healing. It does this by promoting angiogenesis, modulating inflammatory cytokines, and enhancing keratinocyte migration. There are many clinical trials that have reported faster re-epithelialization and improved postoperative or traumatic scar quality [42-51]. PBM provides dual antimicrobial and anti-inflammatory effects for acne management. The combined actions of blue light (415 nm), which targets Cutibacterium acnes through porphyrin-mediated phototoxicity, and red light (630-660 nm), which reduces inflammation and supports lesion resolution, have demonstrated clinical benefit. There are clinical studies that demonstrate PBM’s actions in reducing inflammatory papules, decreasing erythema, and improving post-inflammatory healing [51-59].

There is also clinical evidence supporting PBM as a therapeutic modality for hair restoration. Mitochondrial activation enhances dermal papilla cell function, upregulates growth-related pathways, and prolongs the anagen phase. There are multiple randomized trials that report significant increases in hair density and shaft thickness in androgenetic alopecia using LED-based devices [60-64]. There are more uses for PBM outside of dermatologic applications. PBM effectively reduces musculoskeletal pain and inflammation by modulating oxidative stress, altering cytokine profiles, and improving microcirculation [65-71]. Improvements have also been noted in mitochondrial respiration and bioenergetic function, therefore further supporting PBM’s potential as a systemic therapeutic modality [1,2]. 

Additional studies have explored PBM in conditions such as striae distensae, where enhanced dermal remodeling leads to improved texture and reduced scar visibility [72-74]. PBM continues to gain interest across numerous medical specialties. There are a number of clinical trials showing promising results in the use of PBM in neurological concerns. This includes traumatic brain injury and retinal disorders such as age-related macular degeneration, and neurodegenerative diseases, including Parkinson’s and Alzheimer’s disease [75-78]. PBM has broad therapeutic relevance beyond cosmetic applications. Together, these studies demonstrate consistent short-term improvements in skin texture, collagen density, inflammatory markers, and patient-reported outcomes; however, significant variation in wavelength selection, treatment duration, and outcome measures limits the ability to directly compare across multiple clinical trials and prevents standardized treatment recommendations.

Aesthetic Treatment Protocols

Most clinical trials demonstrate that using RLT for approximately 10-12 minutes a day, twice per week, leads to measurable improvements in collagen production, skin elasticity, and wrinkle reduction within a time frame of roughly 4-12 weeks [26,34,38,79]. Mechanistically, dual-wavelength red and NIR light enhance dermal remodeling by boosting ATP and stimulating fibroblast production of type I/III collagen and elastin, thereby inducing elastin fiber crosslinking. These changes translate to improved firmness and wrinkle reduction over time [80]. However, there is currently no standardized protocol defining the optimal duration or frequency required to achieve long-term, sustained results, highlighting a valuable area for future research. Establishing evidence-based dosing guidelines could improve treatment consistency, maximize clinical outcomes, and support broader integration of RLT into dermatologic practice. While short-term protocols are commonly studied, there is limited evidence regarding how long a patient needs to continue using RLT as maintenance [81]. 

Safety Considerations

Although rare, PBM has been shown to exhibit dose-dependent adverse effects, including erythema and blistering. PBM stimulates an endothelium-derived vasoactive species containing NO within vascular tissue [82]. This molecule enhances vasodilation and can therefore cause an increase in blood flow for up to 30 minutes after treatment. This mechanism explains the other adverse effects noted with photobiomodulation, including discomfort related to heat exposure and erythema due to vasodilation. Patients with higher Fitzpatrick skin types may require careful parameter selection with a lower safety threshold due to differences in light absorption and melanocyte activity [83,84]. Given the potential adverse effects, it is imperative to follow manufacturer recommendations regarding use. One potential concern regarding the use of PBM on the face is adverse effects on vision. However, a preliminary study using PBM for macular degeneration has been shown to be safe and well tolerated, supported by functional and anatomical data [76,85]. Overall, PBM offers a non-toxic approach that is generally well tolerated and has been demonstrated to be low risk for aesthetic purposes [86]. 

Expanding Clinical Indications in Dermatology

Photobiomodulation is increasingly being investigated for dermatologic conditions that extend beyond aesthetic applications. There is emerging evidence supporting the use of RLT in disorders of pigmentation and inflammatory dermatoses, as well as its use in post-procedural recovery. Melasma is a chronic acquired pigmentary disorder marked by patchy facial hyperpigmentation. Melanocytes are melanin-producing cells that are responsible for the hyperpigmented appearance of melasma [87]. It commonly occurs in adult women, particularly those of reproductive age [88,89]. Clinical trials have demonstrated that the use of LED at 585 nm was able to inhibit the maturation of melanocytes, inhibiting melanogenesis from occurring, as well as actively degrading melanin. This acts to reduce vascularization and therefore minimize erythema of the affected area [90]. In the treatment of vitiligo, the mechanism of treatment almost appears to do the opposite compared to the treatment of rosacea. Vitiligo is an acquired pigmentation disorder that is caused by the autoimmune destruction of functional melanocytes. Photobiomodulation therapy, when conducted at 632.8 nm, has the ability to promote repigmentation. This effect is mediated through activation of mitochondrial signaling pathways in melanocyte stem cells and melanoblasts, thereby triggering their differentiation, migration, and functional maturation into pigment-producing melanocytes [91,92].

In the treatment of rosacea, a chronic inflammatory disease characterized by erythema, LED therapy decreases inflammatory cell infiltration by downregulating inflammatory cytokines p65 and S100A9 [93]. It also aids in regulating the activation of TRPV1, which is a receptor that causes the discomfort associated with rosacea, and thereby relieves burning, stinging, and itching [94]. There is also research indicating the use of PBM at 660 nm to help in the recovery of dermal wounds. PBM acts to stimulate the recruitment of pericytes, which coordinate with endothelial cells to stabilize microvessels and regulate local perfusion, thereby leading to accelerated healing [95].

Product selection considerations

Given the wide range of available devices, consumers can utilize the manufacturer’s website to determine which products are effective and safe [96]. Reputable companies will clearly list the wavelengths used, ideally within the effective range of 630-660 nm for red light and 830-850 nm for NIR light, and may provide information about U.S. FDA clearance [97]. Manufacturers should also include links to published studies or clinical trials and disclose the involvement of any medical advisors. Websites that make bold claims without providing scientific references should be interpreted with caution. 

Beyond the company’s materials, consumers can search for independent studies using databases such as Cochrane, ClinicalTrials.gov, PubMed, or Google Scholar. Entering the device or company name along with terms like “LED,” “red light therapy,” or “clinical trial” can help surface any published research. Peer-reviewed studies, particularly randomized controlled trials involving human participants, offer the strongest support for a device’s effectiveness. It is also useful to check the FDA’s 510(k) database, which lists medical devices that have received regulatory clearance in the United States. A listing here indicates that the device has undergone safety and efficacy review and is legally marketed for a specific indication, such as wrinkle reduction or acne treatment.

A literature review was conducted to identify PBM products supported by clinical research. Among the many products available on the market, 10 were found to have studies evaluating the device’s performance in skin rejuvenation. The following section summarizes key considerations for device evaluation and highlights the importance of scientific evidence in guiding product selection. 

Look for Proven Wavelengths 

The most effective devices use red light (around 630-660 nm) and NIR light (around 830-850 nm) [26]. These wavelengths penetrate deeply into the skin, targeting mitochondria to stimulate collagen production, reduce inflammation, and improve healing [96-98]. Pulsed light devices like Gentlewaves® (Light BioScience, LLC, Virginia Beach, Virginia) use a slightly broader range (590-870 nm) and have demonstrated benefits such as reduced inflammation and skin redness [81].

 FDA clearance signals safety and regulatory oversight

Consumers should prioritize FDA-cleared devices when available. The FDA 510(k) database and AccessGUDID.gov may be consulted to verify if a product is FDA-cleared. While a lack of FDA clearance doesn’t mean a product is unsafe, it does mean the device hasn’t been formally evaluated by U.S. regulators, which is particularly relevant for at-home use.

RLT masks are classified as Class II medical devices, a designation indicating a moderate risk to consumers. Other Class II devices include powered wheelchairs and some pregnancy test kits. FDA clearance indicates that the product has been reviewed for safety and efficacy compared to other products already on the market before sale in the United States. 

When analyzing medical devices, it is important to understand the various classifications provided by the FDA. For example, FDA clearance is different from FDA approval, which is reserved for Class III products that pose greater risks, such as pacemakers, breast implants, and ventilators. Table 1 provides a review of FDA terms and pathways of regulation.

**Table 1:** U.S. Food and Drug Administration (FDA) classification of medical devices. FDA: Food and Drug Administration

Classification	Pathway	Risk Class	Regulatory Meaning
FDA-approved	Premarket approval	Class III (high risk)	Proven safe/effective
FDA-cleared	510(k)	Class II (moderate risk)	Substantially equivalent to products already on the market
FDA-registered	510(k)-exempt	Class I (low risk) and some Class II	Registered & compliant
FDA-granted	De novo	Class I or II	First-of-a-kind, low/moderate risk
FDA-authorized	Emergency use authorization	Varies (emergency use)	Temporary use during an emergency
Breakthrough	Breakthrough program	Varies (high impact)	Prioritized review and no approval for life-threatening or irreversibly debilitating diseases or conditions

Any new product sold in the United States with a Classes I, II, or III device classification that does not require a premarket approval application must submit a 510(k) form [99]. Since photobiomodulation masks meet the Class II definition, the FDA’s 510(k) database is a resource that provides premarket safety, equivalence to other products already on the market, and clearance information about these medical devices. The 510(k) database is searchable by entering a specific product name or code and provides a list of all equivalent products. Most red light masks used for anti-aging, wrinkle reduction, and cosmetic appearance fall under the Occupational Health and Safety (OHS) product code. Table 2 includes a list of codes that are associated with photobiomodulation masks.

**Table 2:** U.S. Food and Drug Administration (FDA) product classification for photobiomodulation devices. Class II: moderate-to-high-risk general controls and special controls; OHS: Occupational Health and Safety

Product Code	Device Classification	Application	Regulatory Class	510(k)?
OHS	Light-based over-the-counter wrinkle reduction	Reduce wrinkles on the face, head, and neck	Class II	Yes
ILY	Lamp, infrared, therapeutic heating	Pain relief (muscle and joint), circulation, and inflammation	Class II	Yes
OLP	Over-the-counter powered light-based laser for acne	Mild-to-moderate acne vulgaris	Class II	Yes

An alternative to the 510(k) database is AccessGUDID.gov (Global Unique Device Identification Database). The National Library of Medicine and the FDA have created the database to make comprehensive device information available to anyone. The database includes device information submitted to the FDA, including safety data, storage information, product codes, and FDA submission information. In addition to the FDA product codes, the Global Medical Device Nomenclature (GMDN) Agency provides standard, unique codes for naming and classifying medical devices. These codes provide definitions of medical devices, allowing manufacturers, regulators, and healthcare professionals a common language for medical devices with the goal of improving patient safety on a global level. 

Prioritize peer-reviewed research

Devices with published clinical trials or peer-reviewed studies offer greater credibility. For example, Omnilux® (Omnilux Ltd., Napa, California) was tested in a randomized controlled trial that showed significant wrinkle reduction, improved skin texture, and high user satisfaction [29]. A Korean split-face study on LG Pra. L Mask BWJ1 Derma LED Mask® (LG Electronics Inc., Pyeongtaek, Gyeonggi-do, South Korea) reported improvements in elasticity and hydration with no side effects after eight weeks [38]. Gentlewaves® (Light BioScience, LLC, Virginia Beach, Virginia) has peer-reviewed studies showing its benefits for periorbital wrinkles and inflammation and has obtained FDA clearance [81]. By contrast, some luxury devices may be appealing aesthetically but lack regulatory clearance and large-scale studies, though preliminary results are encouraging [34].

Of the 148 products listed under the OHS product code, only two have associated clinical trials when using the filter available on the FDA database [100]. However, randomized controlled trials have been found on the Cochrane and ClinicalTrials.gov databases for products listed in the 510(k) database that do not populate when searching with the clinical trials filter. It may be necessary to search for the product in other databases to analyze a complete picture of the available data.

All current products on the market with FDA clearance and supporting literature are listed in Table 3. This table is a compilation of FDA-cleared products under the OHS product code that were searched for in the Cochrane, Wiley, PubMed, Embase, and ClinicalTrials.gov databases by device name and separately by device manufacturer. A total of 13 devices were found to have FDA clearance and clinical trials exploring their efficacy. Three of these devices did not meet the scope of this paper and were excluded from analysis. Of the remaining 10 devices, seven have been published in peer-reviewed journals, one has published results directly on the device website, and two refer to studies that are not publicly available.

**Table 3:** Photobiomodulation devices with reported wavelengths, U.S. Food and Drug Administration (FDA) clearance status, and supporting clinical studies. NIR: near-infrared; FDA: Food and Drug Administration; LED: light-emitting diode

Device	Wavelengths	Mode & Application	Literature	FDA 510(k)	Manufacturer
Omnilux® LED System [29]	633 nm red + 830 nm NIR	Clinic‑grade mask with neck and Décolleté attachment	Randomized controlled trial: improved forearm/wrinkle healing	Yes	Omnilux Ltd., Napa, California, United States
Gentlewaves® [81]	590 nm - 870 nm (pulsed)	Clinic-based	Peer-reviewed study: improved periorbital wrinkles and reduced inflammation	Yes	Light BioScience, LLC, Virginia Beach, Virginia, United States
Soli-Tone 2500 LumiFacial® [101]	633 nm + 830 nm	Clinic-based	Pilot study: improved facial appearance and texture in four weeks	Yes	Silhouet-Tone Ltd., Québec, Canada
Neutrogena® [102,103]	445 nm + 630 nm	At-home mask	Evaluator-blinded, randomized study: safe and effective for mild-to-moderate acne. Later recalled and withdrawn [87,88]	Yes	La Lumiere LLC, Neuss, Germany
Silk'n® Home Skin Tightening (HST) [104]	630 ± 20 nm and 850 ± 20 nm	At-home hand-held device	Prospective, single-arm, within-subject (self-controlled) interventional study: improved age-related periorbital wrinkles	Yes	Home Skinovations Ltd., Yokneam, Israel
Celluma® [105,106]	640 nm	At-home mask	Preliminary study published for acne vulgaris. Trial registered with ClinicalTrials.gov for facial wrinkling	Yes	BioPhotas, Inc., Orange County, California, United States
LG Pra.L™ Mask BWJ1 Derma LED Mask [38]	637 nm and 854 nm	At-home mask	Split-face clinical trial: improved elasticity and skin texture	Yes	LG Electronics Inc., Pyeongtaek, Gyeonggi-do, South Korea
TheraFace™ Mask Glo [107]	Not specified	At-home mask	Prospective, interventional, single-arm (monadic), open-label clinical study. Registered on ClinicalTrials.gov. Only published on the device website	Yes	Therabody Inc., Los Angeles, California, United States
Shark® CryoGlow™ [108]	415 nm, 630 nm, and 830 nm	At-home with cryo cooling	Clinical study not publicly available	Yes	SharkNinja Operating LLC, Needham, Massachusetts, United States
CurrentBody® LED Face Mask Series 2 [109]	633 nm, 830 nm, and 1072 nm	At-home mask and neck kit	Clinical study not publicly available	Yes	CurrentBody Ltd., Cheshire, UK

Limitations

Limitations to this review include difficulty finding brand-name devices in the literature. Many clinical trials do not specify the name of the product used, but rather describe the product’s characteristics, making it unclear which device is under investigation. Given that new trials are published continuously, some products that meet the stated criteria may not be included on this list. However, the strategies of researching new products remain consistent and will continue to be a useful tool for device recommendations. Other limitations include recalled products. Neutrogena®’s Mask (La Lumiere LLC, Neuss, Germany) received FDA clearance, had positive supporting clinical trials, but was later recalled due to the theoretical risk of eye injuries from consumer reports of visual effects associated with the mask. Future standardization and transparency in this industry are crucial for both consumers and experts who are certified and licensed to provide medical recommendations on aesthetic devices. The scope of this paper focuses on tips for finding safe and efficacious devices, which leads us to design a narrative review rather than a meta-analysis. Preferred Reporting Items for Systematic Reviews and Meta-Analyses (PRISMA) guidelines have been designed for systematic reviews and meta-analyses and would exclude many of the sources that we have utilized in this review, such as studies that are only published on device manufacturer websites. With this in mind, we have opted not to follow PRISMA guidelines, leading to an inherent bias in our analysis and a lack of specific Boolean search criteria.

Discussion

The explosion of consumer-grade PBM devices, particularly LED masks, has outpaced regulatory and clinical oversight. PBM masks are often promoted with broad claims to appeal to individuals looking for a quick and easy fix to anti-aging. Some manufacturers make claims that exceed the available clinical evidence without offering clear disclosure of treatment parameters or mechanisms of underlying regulatory pathways. A gap remains between marketing and evidence, which does not allow consumers to make an educated decision on which device is best for them. This paper highlights the importance of evaluating devices based on wavelength specificity, clinical trial support, and regulatory classification. Despite variable protocols and limited standardization across studies, high-quality devices have consistently shown measurable benefits and user satisfaction. 

Overall, standardization is needed in the reporting of wavelength, irradiance, fluence, and treatment duration, as well as the total number of sessions among different devices. There needs to be more research aimed at optimizing these treatment parameters and expanding the therapeutic applications of PBM beyond dermatology. Physicians will continue to play a critical role in counseling patients on which devices are safe and backed by clinical evidence. This review aims to provide a framework for both physicians and consumers by addressing the mechanistic data, explaining the clinical evidence, and showing the FDA regulatory pathways. It is important to improve transparency and strengthen the scientific foundation of PBM device recommendations.

## Conclusions

In conclusion, red and NIR light therapy represents a safe, non-invasive approach to improving skin health. As PBM technology becomes more accessible and the market for them continues to expand, it is critical to distinguish between evidence-based devices and unregulated products. Devices with peer-reviewed clinical data and FDA clearance are more likely to achieve meaningful biologic effects while minimizing risk. 

Moving forward, greater standardization in device reporting, dosing protocols, and clinical trial design will be essential to strengthen the scientific foundation of photobiomodulation and improve consistency in outcomes. Physician guidance remains critical in helping patients navigate product selection, set realistic expectations, and use PBM safely and effectively. With all of these factors in play, photobiomodulation can be responsibly integrated into evidence-based dermatologic and aesthetic care with clear therapeutic intent.
